# Prevalence of Cannabinoid Use in Patients With Hip and Knee Osteoarthritis

**DOI:** 10.5435/JAAOSGlobal-D-20-00172

**Published:** 2021-02-02

**Authors:** David G. Deckey, Nina J. Lara, Matthew T. Gulbrandsen, Jeffrey D. Hassebrock, Mark J. Spangehl, Joshua S. Bingham

**Affiliations:** From the Department of Orthopedics, Mayo Clinic Arizona, Phoenix, AZ (Dr. Deckey, Dr. Lara, Dr. Hassebrock, Dr. Spangehl, and Dr. Bingham), and the Department of Orthopedics, Loma Linda Medical Center, Loma Linda, CA (Dr. Gulbrandsen).

## Abstract

**Introduction:**

State legalization and widespread marketing efforts have increased the accessibility and consumption of off-label, non–FDA-approved, cannabinoid (CBD) products. Although clinical evidence is largely absent for the treatment of musculoskeletal pain, patients are experimenting with these products in efforts to relieve joint pain. Assessment of the prevalence, perceived efficacy compared with other nonsurgical modalities, and usage patterns is warranted. The purpose of this study was to report the prevalence and perceived self-efficacy of CBD products in patients with symptomatic hip and/or knee osteoarthritis (OA).

**Methods:**

Two-hundred consecutive patients presenting with painful hip or knee OA were surveyed at their initial evaluation at a large academic center. Using Single Assessment Numeric Evaluation (SANE) scores, survey questions assessed perceived pain and effectiveness of CBD products, in addition to other nonsurgical treatment modalities. Chart review provided demographic factors. Descriptive statistics were used to characterize the data.

**Results:**

Of the 200 patients (80 hip OA, 108 knee OA, and 12 both), 66% were female, and average age was 67 years (range 36 to 89 years). Twenty-four percent (48/200) of patients endorsed use of CBD products before their presentation. The average presenting SANE score (range 0 to 100) for non-CBD users was 50.8 compared with 41.3 among CBD users (*P* = 0.012). Sixty percent of patients learned about CBD through friends, and 67% purchased CBD directly from a dispensary. Oral tinctures (43%) and topical applications (36%) were the most commonly used forms. In addition, 8% of participants in this study had tried marijuana for their pain.

**Conclusion:**

A 24% incidence of CBD usage was found among patients presenting with hip or knee OA. No significant perceived benefit of CBD use seems to exist compared with its nonuse, as patients who used CBD reported significantly worse SANE and visual analogue scale scores than nonusers at baseline. Follow-up studies are warranted to assess these findings.

State legalization and widespread marketing efforts have increased the accessibility and consumption of off-label, non–FDA-approved, cannabinoid (CBD) products. Subsequently, these products have been promoted for the treatment of numerous ailments, including joint pain. Although clinical evidence is largely absent for the treatment of musculoskeletal pain, patients are experimenting with these products in efforts to relieve joint pain.^1,2,3,4,5,6^ If proven effective, these medications could provide multimodal pain control in the treatment of arthritis-related pain.

Surgeons should be aware of the effects of over-the-counter medications, especially non–FDA-approved medications that their patients are consuming. Given the increased availability of CBD products, investigations into the prevalence and perceived efficacy of CBD for treatment of osteoarthritis (OA) are warranted. To our knowledge, data evaluating the prevalence and perceived efficacy of CBD products for the treatment of OA are limited. Therefore, the purpose of this study was to report the prevalence and subjective efficacy of CBD products in patients with symptomatic hip and/or knee OA presenting for an initial orthopaedic surgery consultation.

## Methods

After institutional review board approval, 200 consecutive patients presenting with painful hip or knee OA were surveyed at their initial arthroplasty clinic evaluation at a single high-volume academic center. As part of the initial intake screening, patients were asked to complete a 21-question survey. Questions concerning function and perceived efficacy of treatments were assessed using Single Assessment Numeric Evaluation (SANE) on a 1 to 100 point scale, with a score of 100 indicating the highest perceived benefit (SANE).^7,8^ In addition, medical chart review was undertaken for background demographic factors.

After completion of questionnaires (see appendix for questionnaire example, Appendix 1, http://links.lww.com/JG9/A108), answers were categorized and tabulated. Average SANE scores for interventions were calculated as well. Questions results were binary (yes/no), numeric (SANE/visual analogue scale [VAS]), or free text (ex “Question 14: ‘How did you hear about CBD?’”). Free text answers were manually reviewed for each respondent and categorized into nominal reviewable outcomes (Table 5). Radiographs for every patient were reviewed by two independent reviewers. Descriptive statistics were performed to characterize the population; T-tests were used to compare the variation of continuous variables. Comparison of proportions for sample populations was performed with z-tests. All statistical analysis was performed with JMP statistical software (SAS Institute).

## Results

Of the 200 consecutive patients, 100% completed the survey. Sixty-six percent were female, and the average age was 67 years. Knee OA was the most common complaint (n = 108) followed by hip OA (n = 80), and a minority of patients had symptoms in both joints at presentation (n = 12). Thirty-seven percent of these patients were symptomatic on the right side, 31% on the left side, and 32% presented with bilateral complaints. Knee OA had an average Kellgren-Lawrence OA grade of 2.7 (range 0 to 4). Average Tönnis scale grading of the affected hip OA was 1.8 (range 0 to 3) (Table 1).

**Table 1:** Demographic and Radiographic Variables of Arthroplasty Clinic Sample Population

No. of patients, n	200
Age (y) (±SD)	67.21
Female, n (%)	112 (56)
Joints, n (%)	
Knee	108 (54)
Hip	80 (40)
Both	12 (6)
Laterality, n (%)	
Left	62 (31)
Right	74 (37)
Both	64 (32)
Knee osteoarthritis grade^a^ (n = 159), n (%)	
0	2 (1.1)
1	29 (18.3)
2	34 (21.4)
3	42 (26.4)
4	52 (32.7)
Hip osteoarthritis grade^b^ (n = 107), n (%)	
0	7 (6.5)
1	36 (33.6)
2	31 (29.1)
3	33 (30.8)

a Kellgren-Lawrence grading scale for knee osteoarthritis.

b Tönnis grading scale for hip osteoarthritis.

Twenty-four percent (48/200) of patients endorsed use of CBD products before their presentation. The average presenting SANE score (range 0 to 100) for non-CBD users was 50.8 compared with 41.3 among CBD users (*P* = 0.012). The average VAS score (range 0 to 10) for non-CBD users was 5.7 compared with 6.6 among CBD users (*P* = 0.036). No difference in the asymptomatic contralateral joint SANE score (range 0 to 100) was found when comparing non-CBD users with CBD users (81.9 versus 75.9, respectively, *P* = 0.129) (Table 2).

**Table 2:** SANE and VAS Scores Among Non-CBD and CBD Users, Respectively

Factor	Non-CBD Users (n = 152), n (%)	CBD Users (n = 48), n (%)	*P* Value
Symptomatic joint SANE (average)	50.8	41.3	0.012
Contralateral unaffected joint SANE (average)	81.9	75.9	0.129
VAS pain rating (average)	5.7	6.6	0.036

CBD = cannabinoid, SANE = Single Assessment Numeric Evaluation, VAS = Visual Analogue Scale

Among non-CBD users, 73% had tried NSAIDs for symptomatic relief compared with 90% among the CBD using group. A statistically higher percentage of patients in the CBD group had used NSAIDs for symptomatic relief compared with non-CBD users (*P* = 0.017). No significant difference was found in the number of patients who had tried bracing treatment, steroid injections, or viscosupplementation injections between the two groups. A significantly higher percentage of marijuana use was found among the CBD group compared with non-CBD users (31% versus 1%, respectively, *P* < 0.001) despite similar rates of “Other” recreational drug use (15% CBD users versus 11% non-CBD users) (Table 3).

**Table 3:** Frequency of Alternative Treatments for Symptomatic Osteoarthritis Used by Study Sample Population Non-Cannabinoid (CBD) and CBD Users, Respectively

Factor	Non-CBD Users (n = 152), n (%)	CBD Users (n = 48), n (%)	*P* Value^a^
NSAID	111 (73)	43 (90)	0.017
Bracing treatment	43 (28)	26 (54)	0.289
Steroid injection	79 (52)	28 (58)	0.119
Viscosupplementation injection	30 (20)	11 (23)	0.575
Marijuana	2 (1)	15 (31)	<0.001
Recreational “other” drug use	16 (11)	7 (15)	0.928

a Computed using z-test for difference in proportions.

A significant difference was seen after NSAID use; non-CBD users reported an improvement with an increase in the average SANE to 52.7, whereas CBD users decreased to a SANE of 39.0 (*P* = 0.012). Otherwise, the differences in SANE scores between the two groups after bracing treatment, steroid injection, viscosupplementation injection, or marijuana use were not statistically significant (Table 4).

**Table 4:** SANE Score Averages Among Two Groups After Nonsurgical Treatments

Average SANE Scores	Non-CBD Users (n = 152), n	CBD Users (n = 48), n	*P* Value
Baseline	50.8	41.3	0.012
Post-NSAID	52.7	39.0	0.012
Post–bracing treatment	40.2	37.6	0.727
Post-steroid	54.9	45.9	0.205
Post-viscosupplementation	55.0	43.4	0.225
Post-marijuana	25.0	47.0	0.319

CBD = cannabinoid, SANE = Single Assessment Numeric Evaluation

Among CBD users, 60% of patients learned about CBD through friends, and 67% purchased CBD directly from a dispensary. Oral tinctures (43%) and topical applications (36%) were the most commonly used forms of CBD. Twenty-two percent of all the patients in this sample reported ongoing CBD utilization (Table 5).

**Table 5:** Characterization of CBD Use and Procurement Among the Sample Population

Descriptor	N
Referral source	
HCP	7
Friend	31
Advertisement	13
Work	1
Purchasing location	
HCP	1
Friend	3
Online	10
Store	29
CBD type	
Capsule	5
Topical	16
Oil tincture	19
Edible	4
Frequency of use	
Daily	13
Twice daily	6
Three times daily	2
As needed	19
Only once	4

CBD = cannabinoid, HCP = healthcare provider

## Discussion

In this prospective cohort of 200 consecutive patients, 24% (48 patients) reported trying CBD-containing products for relief of their arthritis-related pain before their initial orthopaedic surgical consultation. Although CBD use has not been previously characterized in this population, its prevalence is similar to the reported 15% to 22% of the general US population that reported marijuana use.^9,10^ However, this reported CBD use is much higher compared with marijuana use in an older population. Han and Palamar^11^ found that 9% of adults aged 50 to 64 years and 2.9% of adults aged 65 years and older reported marijuana use, which was similar to the 9% of patients who reported marijuana use in our study. This large difference in CBD and marijuana usage in a similarly aged population demonstrates the growing trend and popularity of CBD utilization. Given that more and more patients will arrive in clinic having tried or wanting to try these products, it is crucial that the orthopaedic surgeon is aware of CBD products and current trends in utilization. In addition, in the setting of the opioid crisis, it is imperative that we continue to identify new and potentially less-addictive modalities for pain relief. The goal of this study was to characterize and analyze CBD usage and perceived effectiveness in patients presenting for primary consultation with hip and/or knee OA.

To understand why CBD has become such a rapidly growing trend, a brief history is helpful. The passage of the US Hemp Farming Act of 2018 removed hemp (defined as cannabis with less than 0.3% tetrahydrocannabinol [THC]) from Schedule I Controlled Substances.^12^ CBD can be derived from cannabis, which comes from the plant *Cannabis sativa*. Virtually overnight, a new US industry was created. This industry brought with it a legal, unregulated product with broad claims of treating anxiety, insomnia, PTSD, and reducing pain and inflammation. Although not containing high percentages of THC, hemp can still contain CBD, which augments the body's endogenous CBD system primarily through CB1 and CB2 receptors in both the central and peripheral nervous system. These receptors have been shown to play roles in modulating nociception and inflammatory pathways.^13^ However, the full effects of CBD are still not fully understood. Although animal models have shown CBD to decrease OA-related pain,^14,15,16,17,18,19^ its efficacy in humans has not been fully supported.^18,20,21^

As the stigma surrounding THC and CBD use decreases and these products become more readily available, the prevalence of their use will likely increase. Previously, research has been hampered by lack of funding and the Schedule I classification of cannabis. Given the wide availability of CBD in the United States at present and movements to remove cannabis from the Schedule I classification, it is believed that more knowledge about how THC/CBD functions will come to light. A study using National Inpatient Sample database showed that marijuana/THC use was associated with decreased mortality in patients undergoing total hip arthroplasty (THA), total knee arthroplasty (TKA), total shoulder arthroplasty (TSA), and traumatic femur fixation.^22^ In addition, two previous, recently published studies in the orthopaedic literature have explored the use of CBD and THC in arthroplasty.^4,5^ Hickernell et al^4^ examined the use of dronabinol, a synthetic form of THC, in a multimodal pain regimen after THA and TKA surgery. In their study, the group taking a prescribed dose of drocannabinol had significantly shorter stays and significantly fewer total morphine equivalents. However, this was a small (81 patients) retrospective study and warrants further studies to fully support this trend. Runner et al^5^ found that 16.4% of patients following TKA or THA reported use of CBD or THC in the perioperative period. Compared with nonusers, no significant difference was observed in the length of narcotic use, total morphine equivalents used, postoperative pain scores, or the length of stay. Patients in this study were self-medicating without uniformity, which is in contrast to the prescribed dose of drocannabinol used in the Hickernell study.

Our study, however, showed no significant perceived benefit of CBD use compared with nonuse, and patients who used CBD actually reported significantly worse SANE and VAS scores at baseline than nonusers. The symptomatic joint(s)' SANE score significantly differed between CBD users and nonusers at initial presentation (41.3 versus 50.8, *P* = 0.012). Previous literature has suggested that the minimally clinically important difference for knee injury interventions is approximately 7 to 19, suggesting that perhaps baseline presentation SANE scores may have been statistically different but not clinically measurable.^23^ In addition, VAS pain rating for CBD users was significantly higher at baseline than nonusers (6.6 versus 5.7, *P* = 0.036). Interestingly, patients who used CBD products were also significantly more likely to use NSAIDs. This finding suggests that the patients taking CBD products may have had more symptomatic OA or more prone to self-medicating. Patients who reported CBD use were also significantly more likely to report marijuana use.

Several limitations of this study must be acknowledged. Although this was a prospective study, recall bias may be present as patients were asked to recall use of treatment and its effectiveness leading up to their first visit. In addition, only patients presenting for primary hip and knee arthroplasty consultation were included in this study. This restriction limits the generalizability of our findings to other orthopeadic specialties. Future studies are warranted in other subspecialties, such as sports medicine, where injuries are more acute. The perceived efficacy of CBD products may be different for acute pain than for chronic pain. The source of CBD product and route of administration was also not standardized, which may play a role in its effectiveness. In addition, this study had a limited sample size of 200 patients and as such may be subject to type 2 error when concluding no difference. Therefore larger, multicenter studies are needed to fully evaluate CBD use in this population and to enhance generalizability as well as a randomized controlled trial with placebo and a controlled dose of CBD. Finally, a substratification of severity of OA in either group would be useful in future studies attempting to determine the efficacy of CBD in symptomatic relief.

## Conclusion

To our knowledge, this is the first prospective study to evaluate the usage of over-the-counter CBD products in a hip and knee OA population. A 24% incidence of CBD usage was found among these patients. We found no significant perceived benefit of CBD use compared with nonuse, and patients who used CBD actually reported significantly worse SANE and VAS scores than nonusers.
